# Entropy as a Topological Operad Derivation

**DOI:** 10.3390/e23091195

**Published:** 2021-09-09

**Authors:** Tai-Danae Bradley

**Affiliations:** Sandbox@Alphabet, Mountain View, CA 94043, USA; tai.danae@math3ma.com

**Keywords:** Shannon entropy, topology, operad

## Abstract

We share a small connection between information theory, algebra, and topology—namely, a correspondence between Shannon entropy and derivations of the operad of topological simplices. We begin with a brief review of operads and their representations with topological simplices and the real line as the main example. We then give a general definition for a derivation of an operad in any category with values in an abelian bimodule over the operad. The main result is that Shannon entropy defines a derivation of the operad of topological simplices, and that for every derivation of this operad there exists a point at which it is given by a constant multiple of Shannon entropy. We show this is compatible with, and relies heavily on, a well-known characterization of entropy given by Faddeev in 1956 and a recent variation given by Leinster.

## 1. Introduction

In this article, we describe a simple connection between information theory, algebra, and topology. To motivate the idea, consider the function d:[0,1]→R defined by

$$d\left(x\right)=\left\{\begin{array}{cc} -x\log x & \mathrm{if}\;x>0,\\ 0 & \mathrm{if}\;x=0. \end{array}\right.$$

This map satisfies an equation reminiscent of the Leibniz rule from Calculus, d(xy)=d(x)y+xd(y) for all x,y∈[0,1]. In other words, *d* is a nonlinear derivation [1], (Lemma 2.2.6). This derivation may also bring to mind the Shannon entropy of a probability distribution. Indeed, a probability distribution on a finite set {1,…,n} for n≥1 is a tuple of nonnegative real numbers $p=(p_{1},\ldots ,p_{n})$ satisfying $\sum_{i=1}^{n}p_{i}=1$, and the *Shannon entropy* of *p* is defined to be

$$H\left(p\right)=-\sum_{i=1}^{n}p_{i}\log p_{i}=\sum_{i=1}^{n}d\left(p_{i}\right).$$

Although *d* is not linear, this may prompt one to wonder about settings in which Shannon entropy itself is a derivation. We describe one such setting below by showing a correspondence between Shannon entropy and derivations of the operad of topological simplices.

### 1.1. Motivation

As evidenced by recent work, the intersection of information theory and algebraic topology is fertile ground. In 2015 tools of information cohomology were introduced in [2] by Baudot and Bennequin who construct a certain cochain complex for which entropy represents the unique cocycle in degree 1. In the same year, Elbaz-Vincent and Gangl approached entropy from an algebraic perspective and showed that what are known as information functions of degree 1 behave “a lot like certain derivations” [3]. A few years prior in 2011, Baez, Fritz, and Leinster gave a category theoretical characterization of entropy in [4], which was recently extended to the quantum setting by Parzygnat in [5]. In preparation of that 2011 result, Baez remarked in the informal article [6] that entropy appears to behave similarly to a derivation in a certain operadic context, an observation we verify and make explicit below. Cohomological ideas are also explored in Mainiero’s recent work, where entropy is found to appear in the Euler characteristic of a particular cochain complex associated to a quantum state [7]. Upon taking inventory, one thus has the sense that entropy behaves somewhat similar to “*d* of something,” for some (co)boundary-like operator d. The present article is in this same vein. Notably, once a few simple definitions are in place, the mathematics is quite straightforward. Even so, we feel it is worth sharing if for no other reason than to provide a glimpse at yet another algebraic and topological facet of entropy.

### 1.2. Background

To start, our work is based on a particular characterization of Shannon entropy that is compatible with an operadic viewpoint. Let $\Delta^{n}$ denote the standard topological *n*-simplex for n≥0,

$$\Delta^{n}:=\left\{\left(p_{0},p_{1},\ldots ,p_{n}\right)\in R^{n+1}|0\leq p_{i}\leq 1\;\mathrm{and}\;\sum_{i=0}^{n}p_{i}=1\right\},$$

where $\Delta^{0}$ denotes the unique probability distribution on the one-point set. More generally, any probability distribution $p=(p_{0},\ldots ,p_{n})$ on an n+1-element set is a point in $\Delta^{n}$. Given n+1 probability distributions $q^{i}=\left(q_{0}^{i},\ldots ,q_{k_{i}}^{i}\right)\in \Delta^{k_{i}}$ where i=0,1,…,n, they may be composed with *p* simultaneously to obtain a point in $\Delta^{k_{0}+k_{1}+\cdots +k_{n}+n}$ denoted by

$$p\circ \left(q^{0},q^{1},\ldots ,q^{n}\right):=\left(p_{0}q_{0}^{0},\ldots ,p_{0}q_{k_{0}}^{0},p_{1}q_{1}^{1},\ldots ,p_{1}q_{k_{1}}^{1},\ldots ,p_{n}q_{1}^{n},\ldots ,p_{n}q_{k_{n}}^{n}\right).$$

As shown in [1] and reviewed below, this composition of probabilities finds a natural home in the language of operads. Furthermore, it plays a key role in a well-known 1956 characterization of Shannon entropy due to D. K. Faddeev [8]. A proof of a slight variation of Faddeev’s result was recently given by Leinster [1], (Theorem 2.5.1). That is the version we quote here.

**Theorem** **1** (Faddeev-Leinster). *Let ${\left\{F:\Delta^{n}\to R\right\}}_{n\geq 0}$ be a sequence of functions. The following are equivalent:*

*1.*  *the functions F are continuous and satisfy*
  (1) $$F\left(p\circ \left(q^{0},\ldots ,q^{n}\right)\right)=F\left(p\right)+\sum_{i=0}^{n}p_{i}F\left(q^{i}\right)$$
  *where n≥0 and $p\in \Delta^{n}$ and $q^{i}\in \Delta^{k_{i}}$ with $k_{0}$, $k_{1},\ldots ,k_{n}\geq 0$;*
*2.*  *F=cH for some c∈R.*

To make the connection with derivations, let us introduce some notation. Given a probability distribution $p\in \Delta^{n}$ let $\bar{p}:R^{n+1}\to R$ denote the function that maps a point $x=(x_{0},\ldots ,x_{n})$ to the standard inner product $\langle p,x\rangle =\sum_{i=0}^{n}p_{i}x_{i}$. Then, when F=H, Equation (1) may be rewritten as

(2) $$H\left(p\circ \left(q^{0},\ldots ,q^{n}\right)\right)=H\left(p\right)+\bar{p}\left(H\left(q^{0}\right),\ldots ,H\left(q^{n}\right)\right).$$

This equation is one hint that entropy might be a derivation, although a “*q*” is notably absent from the first term on the right-hand side. As a further teaser, Baez explored an algebraic interpretation of Equation (2) in the informal article [6], where the reader is reminded that Shannon entropy is a derivative of the partition function of a probability distribution with respect to Boltzmann’s constant, considered as a formal parameter. In that article, Equation (2) follows in a few short lines from this computation. One is thus motivated to look for a general framework of operad derivations for which Equation (2) is an example. This is what we describe below.

Section 2 reviews the definition of operads and representations of them. We will recall that the collection of topological simplices admits the structure of an operad as in [1] and that R gives rise to a representation of it. In Section 3, we define an abelian bimodule *M* over any operad O and the notion of a derivation of O with values in *M*. With these definitions in place, Equation (2) will find a generalization in Proposition 1, and the main result will quickly follow.

**Theorem** **2.** *Shannon entropy defines a derivation of the operad of topological simplices, and for every derivation of this operad there exists a point at which it is given by a constant multiple of Shannon entropy.*

## 2. Background: Operads and Their Representations

**Figure 1:**
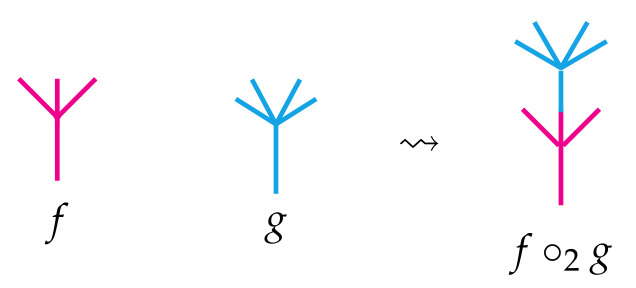
One of the three ways to compose a 4-ary operation *g* with a 3-ary operation *f*.

In an introduction to operads, it is helpful to first think about algebras. An algebra *A* is a vector space *V* equipped with a bilinear map μ:V×V→V thought of as multiplication. Depending on whether μ satisfies a particular relation, the algebra will usually be described by an approriate qualifier. For instance, if μ(v,w)=μ(w,v) for all v,w∈V, then *A* is called a *commutative algebra*; if μ(μ(u,v),w)=μ(u,μ(v,w)) for all u,v,w∈V, then *A* is a called an *associative algebra*, and so on. Behind each of these algebras is a particular operad that encodes the behavior of the multiplication map μ. To motivate the formal definition, it is helpful to visualize μ as a planar binary rooted tree and more generally to imagine an arbitrary *n*-ary operation as a planar rooted tree with *n* leaves. There is a natural way to compose such operations. For instance, when *f* is a 3-ary operation and *g* is a 4-ary operation, they may be composed to obtain a 6-ary operation by using the output of *g* as one of the inputs of *f* as illustrated in Figure 1. There *g* has been grafted into the *second* leaf of the tree associated to *f*, and so we denote that choice with the subscript “$\circ_{2}$” in the figure. There are two other composites $f\circ_{1}g$ and $f\circ_{3}g$, which are not shown but are obtained similarly.

In general, there are *n* ways to compose an *m*-ary operation with an *n*-ary operation, and the resulting operation will always have arity m+n−1. This composition should further satisfy some sensible associativity and unital axioms, and the collection of all such operations with their compositions is called an *operad*. The concept has origins in category theory [9] and has been used extensively in algebraic topology and homotopy theory [10,11,12,13,14] with applications in physics as well [15,16]. Operads may be defined in any symmetric monoidal category, and for ease of exposition below, we will assume all categories C are concrete (that is, all objects have underlying sets) so that we may refer to *elements* in a given object of C. Indeed, the main example to have in mind is the category of topological spaces.

**Definition** **1.** *Let C be a symmetric monoidal category with monoidal product ⊗.. An*operad*in C consists of a sequence of objects {O(1),O(2),…} together with morphisms*

$$\circ_{i}:O\left(n\right)⊗O\left(m\right)\to O\left(n+m-1\right)$$

*in C for all n,m≥1 and 1≤i≤n and an operation 1∈O(1) satisfying the following:*

*(i)*  *[associativity] For all p∈O(n) and q∈O(m) and r∈O(k),*
$$\begin{array}{c} \left(p\circ_{j}q\right)\circ_{i}r=\left\{\begin{array}{cc} \left(p\circ_{i}r\right)\circ_{j+k-1}q & \mathrm{if}\;1\leq i\leq j-1\\ p\circ_{j}\left(q\circ_{i-j+1}r\right) & \mathrm{if}\;j\leq i\leq j+m-1\\ \left(p\circ_{i-m+1}r\right)\circ_{j}q & \mathrm{if}\;i\geq j+m \end{array}\right. \end{array}$$
*(ii)*  *[identity] The operation 1∈O(1) acts as an identity in the sense that*
$$1\circ_{1}p=p\circ_{i}1=p$$
*for all p∈O(n) and 1≤i≤n.*

**Figure 2:**
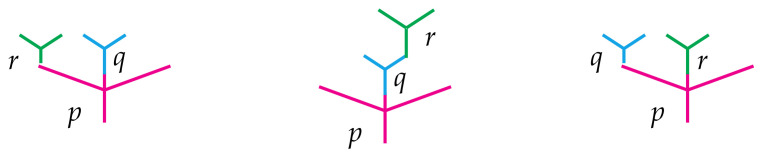
Associativity in an operad. (**Left**) First composing *q* with *p* and then *r* is the same as first composing *r* with *p* and then *q*. The order in which this is performed does not matter. (**Right**) The same is true if *r* appears to the right, rather than the left, of q. (**Middle**) Likewise, *r* may first be composed with *q* and their composite may then be composed with *p*, or *q* may be first composed with *p* followed by r. Again, the order does not matter.

The definition is conceptually simple despite its cumbersome appearance. For instance, Figure 2 illustrates the associativity requirements listed in item (i).

As mentioned above, one often thinks of the elements O(n) as abstract *n*-to-1 operations, and the morphisms $\circ_{i}$ specify a way to compose them. It is common to begin indexing the sequence of objects at n=0 to account for 0-ary operations, but as we will soon see, our main example of an operad in Example 2 will have no 0-ary operations, and so our definition starts with O(1). We do not consider an action of the symmetric group and so O is sometimes called a *non-symmetric operad*, but we will simply call it an operad. In the special case when C is the category of vector spaces with linear maps and ⊗ is the tensor product, O is often called a *linear operad*. When it is the category Top of topological spaces with continuous maps and ⊗ is the Cartesian product, O is often called a *topological operad*.

**Example** **1.** *Given a set X, the*endomorphism operad*is ${\mathrm{End}}_{X}=\left\{{\mathrm{End}}_{X}\left(1\right),{\mathrm{End}}_{X}\left(2\right),\ldots \right\}$ where ${\mathrm{End}}_{X}\left(n\right):=C\left(X^{n},X\right)$ denotes the set of all functions from the n-fold Cartesian product $X^{n}$ to X. The unit operation in ${\mathrm{End}}_{X}\left(1\right)$ is the identity function ${\mathrm{id}}_{X}:X\to X.$ If $f\in C(X^{n},X)$ and $g\in C(X^{m},X)$ are a pair of functions, then for each i=1,…,n the composition $f\circ_{i}g$ is obtained by using the output of g as the ith input of f. Explicitly, given $\left(x_{1},\ldots ,x_{n+m-1}\right)\in \;X^{n+m-1}$,*

$$\left(f\circ_{i}g\right)\left(x_{1},\ldots ,x_{n+m-1}\right):=f\left(x_{1},\ldots ,x_{i-1},g\left(x_{i},\ldots ,x_{i+m-1}\right),x_{i+m},\ldots ,x_{n+m-1}\right).$$

*The simultaneous composition of several functions may also be considered. That is, given n functions $g_{i}\in C\left(X^{k_{i}},X\right)$ where i=1,…,n they may be composed with f simultaneously to obtain a new function $f\circ \left(g_{1},\ldots ,g_{n}\right)\in C\left(X^{k_{1}+\cdots +k_{n}},X\right)$, which is again defined by using the outputs of the $g_{i}$ as the inputs of f. Explicitly, given $\left(x_{1},\ldots ,x_{k_{1}+\cdots +k_{n}}\right)\in X^{k_{1}+\cdots +k_{n}}$, we have*

$$\left(f\circ \left(g_{1},\ldots ,g_{n}\right)\right)\left(x_{1},\ldots ,x_{k_{1}+\cdots +k_{n}}\right)=f\left(g_{1}\left(x_{1},\ldots ,x_{k_{1}}\right),\ldots ,g_{n}\left(x_{k_{1}+\cdots +k_{n-1}+1},\ldots ,x_{k_{1}+\cdots +k_{n}}\right)\right)$$

**Example** **2.** *The simplices $\Delta^{0},\Delta^{1},\Delta^{2},\ldots$ give rise to a topological operad called*the operad of topological simplices*$\Delta =\{\Delta_{1},\Delta_{2},\ldots \}$ where $\Delta_{n}:=\Delta^{n-1}$. The unit operation in $\Delta_{1}$ is the unique probability distribution on a one-point set. If $p=\left(p_{1},\ldots ,p_{n}\right)\in \Delta_{n}$ and $q=\left(q_{1},\ldots ,q_{m}\right)\in \Delta_{m}$ are probability distributions, then the composition $p\circ_{i}q$ is obtained by multiplying each of the m coordinates of q by $p_{i}$ and then replacing the ith coordinate of p with the resulting m-tuple. Explicitly,*

$$p\circ_{i}q:=\left(p_{1},\ldots ,p_{i}q_{1},\ldots ,p_{i}q_{m},\ldots ,p_{n}\right)\in \Delta_{n+m-1}.$$

*Equivalently, the distribution p may be visualized as a planar tree with n leaves labeled by the probabilities $p_{1},\ldots ,p_{n}$ and similarly for q. Then the composition $p\circ_{i}q$ is obtained by “painting” each of the leaves of q with the probability $p_{i}$ and grafting the resulting tree into the ith leaf of p as below. Notice the sum of the probabilities on the leaves on the composite tree is 1.* 
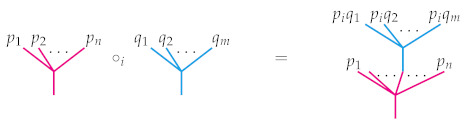

**Figure 3:**
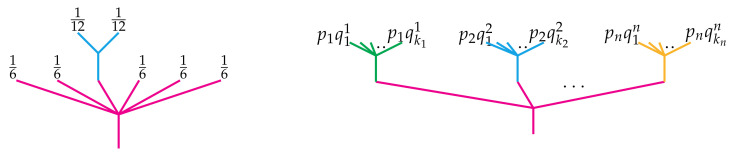
(**Left**) A picture of the composition $p\circ_{3}q$ when *p* is the probability distribution associated to a six-sided die and *q* is that of a fair coin toss. (**Right**) The simultaneous composition of *n* probability distributions $q^{i}\in \Delta_{k_{i}}$ with a given $p\in \Delta_{n}$.

*As an example, if $p=\left(\frac{1}{6},\ldots ,\frac{1}{6}\right)$ represents the probability distribution of rolling a six-sided die and $q=\left(\frac{1}{2},\frac{1}{2}\right)$ is that of a fair coin toss, then $p\circ_{3}q=\left(\frac{1}{6},\frac{1}{6},\frac{1}{12},\frac{1}{12},\frac{1}{6},\frac{1}{6},\frac{1}{6}\right)$ is a point in $\Delta_{7}$, whose picture is shown on the left of Figure 3.*

*Further recall that if we have n different distributions $q^{i}=\left(q_{1}^{i},\ldots ,q_{k_{i}}^{i}\right)\in \Delta_{k_{i}}$ where i=1,…,n, then we may compose them with p simultaneously to obtain the following point in $\Delta_{k_{1}+\cdots +k_{n}},$*

$$p\circ \left(q^{1},\ldots ,q^{n}\right)=\left(p_{1}q_{1}^{1},\ldots ,p_{1}q_{k_{1}}^{1},p_{2}q_{1}^{2},\ldots ,p_{2}q_{k_{2}}^{2},\ldots ,p_{n}q_{1}^{n},\ldots ,p_{n}q_{k_{n}}^{n}\right).$$

*This simultaneous composition is illustrated by the tree on the right in Figure 3.*

Just as groups come to life when considering representations of them, so operads come to life when each abstract *n*-ary operation is mapped to a concrete *n*-ary operation on a particular object. This assignment is traditionally called an *algebra* of the operad, but we prefer the more descriptive name *representation.*

**Definition** **2.** *Let O be an operad in the category of sets. A*representation of O*, or an*O-representation*, is set X together with functions*

$$\varphi_{n}:O\left(n\right)\to {\mathrm{End}}_{X}\left(n\right)\;\mathrm{for}\;n\geq 1$$

*that respect the operad unit and compositions. That is, $\varphi_{n}\left(1\right)=1$ and*

$$\varphi_{n+m-1}\left(p\circ_{i}q\right)=\varphi_{n}\left(p\right)\circ_{i}\varphi_{m}\left(q\right)$$

*for all p∈O(n),q∈O(m) and 1≤i≤n.*

Importantly, one may also wish to define a representation of an operad in any symmetric monoidal category C whenever “${\mathrm{End}}_{X}\left(n\right)$” is in fact an object in C. It must consist of an object *X* together with a family of morphisms $O\left(n\right)\to {\mathrm{End}}_{X}\left(n\right)$ in C that are compatible with the operad unit and compositions. This holds, for instance, when the monoidal category C is also closed—that is, when it is equipped with an internal hom functor that is compatible with the monoidal product. Monoidal closure, however, will not be required in our work, which primarily concerns the category Top of topological spaces. Indeed, the main example to have in mind is when O=Δ is the operad of simplices and X=R is the real line in Top. In this case, we define ${\mathrm{End}}_{R}\left(n\right):=\mathrm{Top}\left(R^{n},R\right)$ to be the space of continuous functions $R^{n}\to R$ equipped with the product topology. Now, consider the continuous maps $\varphi_{n}:\Delta_{n}\to {\mathrm{End}}_{R}\left(n\right)$ given by $p\mapsto \varphi_{n}\left(p\right)$ where $\varphi_{n}\left(p\right)\left(x\right):=\langle p,x\rangle =\sum_{i=1}^{n}p_{i}x_{i}$ whenever $x=\left(x_{1},\ldots ,x_{n}\right)\in R^{n}$. Then, it is simple to check that $\varphi_{n+m-1}\left(p\circ_{i}q\right)=\varphi_{n}\left(p\right)\circ_{i}\varphi_{m}\left(q\right)$ for all p,q, and *i* and that $\varphi_{n}\left(1\right)=1$ for all *n*, and so R is a representation of Δ.

## 3. Derivations of the Operad of Simplices

With these basic definitions in hand, the present goal is to define a mapping *d* out of the topological operad Δ that satisfies an appropriate version of the Leibniz rule,

(3) $$\begin{array}{c} d\left(p\circ_{i}q\right)=dp\circ_{i}q+p\circ_{i}dq\;\left(\mathrm{desideratum}\right) \end{array}$$

for all $p\in \Delta_{n}$ and $q\in \Delta_{m}$ and for all 1≤i≤n. This desired equation suggests the codomain of *d* should be a (bi)module over Δ that is, moreover, an abelian monoid. This motivates the following two definitions, the first of which is a slight generalization of that given by Markl in [15].

**Definition** **3.** *Let O={O(1),O(2),…} be an operad in a symmetric monoidal category C. A*bimodule over O*, or simply an*O-bimodule*, is a collection of objects M={M(1),M(2),…} in C together with morphisms*

$$\begin{array}{cc} \circ_{i}^{L} & =O(n)⊗M(m)\to M(n+m-1)\;(\mathrm{left}\;\mathrm{composition})\\ \circ_{i}^{R} & =M(n)⊗O(m)\to M(n+m-1)\;(\mathrm{right}\;\mathrm{composition}) \end{array}$$

*in C for each 1≤i≤n such that whenever*

$$p⊗q⊗r\in \left\{\begin{array}{c} M(n)⊗O(m)⊗O(k),\mathrm{or}\\ O(n)⊗M(m)⊗O(k),\mathrm{or}\\ O(n)⊗O(m)⊗M(k) \end{array}\right.$$

*the following holds:*

(4) $$\begin{array}{c} \left(p\circ_{j}q\right)\circ_{i}r=\left\{\begin{array}{cc} \left(p\circ_{i}r\right)\circ_{j+k-1}q & \mathrm{if}\;1\leq i\leq j-1\\ p\circ_{j}\left(q\circ_{i-j+1}r\right) & \mathrm{if}\;j\leq i\leq j+m-1\\ \left(p\circ_{i-m+1}r\right)\circ_{j}q & \mathrm{if}\;i\geq j+m. \end{array}\right. \end{array}$$

The associativity requirements displayed in Equation (4)—and hence the intuition behind them—are completely analogous to those defining operads as illustrated in Figure 2. The only difference here is that one of the three operations may come from the bimodule rather than the operad. Here is the main example to have in mind.

**Example** **3.** *As every algebra is a bimodule over itself, so every representation of O is an O-bimodule in a straightforward way. Indeed, in the case of the topological operad of simplices, the maps comprising the *Δ*-representation structure on R induce a *Δ*-bimodule structure on ${\mathrm{End}}_{R}$. However, we will make use of a slight variant of this bimodule structure. Right composition will be defined in the expected way, though left composition will not. Explicitly, we define the left and right composition maps*

$$\begin{array}{cc} \circ_{i}^{L}:\Delta_{n}\times \mathrm{Top}\left(R^{m},R\right) & ⟶\mathrm{Top}(R^{n+m-1},R)\\ \circ_{i}^{R}:\mathrm{Top}\left(R^{n},R\right)\times \Delta_{m} & ⟶\mathrm{Top}(R^{n+m-1},R) \end{array}$$

*as follows. Given a probability distribution *$p\in \Delta_{n}$*and a continuous function*$f:R^{m}\to R$*, define left composition by *$p\circ_{i}^{L}f:=\bar{p}\circ \left(0,\ldots ,0,f,0,\ldots ,0\right)$*, where the composition on the right-hand side is defined as in the simultaneous composition in the endomorphism operad of *R* illustrated in Example 1, and where each 0 denotes the zero function *R→R*. Here, recall that *$\bar{p}:R^{n}\to R$* maps a point x to the standard inner product *〈p,x〉*as introduced in Section 1. Unwinding this, left composition thus evaluates explicitly as *$\left(p\circ_{i}^{L}f\right)\left(x_{1},\ldots ,x_{n+m-1}\right)=p_{i}f\left(x_{i},\ldots ,x_{i+m-1}\right)$*. In words, the value of the left composite *$p\circ_{i}^{L}f:R^{n+m-1}\to R$* at a point x is computed by evaluating f at the m-subtuple of x beginning at the *ith*coordinate and scaling that output by *$p_{i}$*. All other coordinates of x are ignored. The picture to have in mind is that below, where the bold dots are imagined to be “plugs” that prevent the surplus coordinates from playing a role. In this picture,*n=3*and*m=2. 
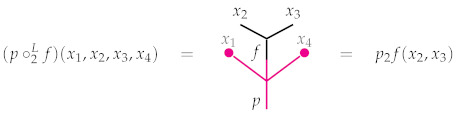

*Given a probability distribution $q\in \Delta_{m}$ and a continuous function $g:R^{n}\to R$, define right composition by*

$$\begin{array}{cc} \left(g\circ_{i}^{R}q\right)(x_{1},\ldots , & x_{n+m-1})\\ \; & :=g(x_{1},\ldots ,x_{i-1},\sum_{k=1}^{m}q_{k}x_{i+k-1},x_{i+m},\ldots ,x_{n+m-1}). \end{array}$$

*This may be understood visually as well. The value of the right composite $g\circ_{i}^{R}q:R^{n+m-1}\to R$ at a point x is computed by taking the inner product of q with the m-tuple of x beginning at the ith coordinate and using that number as the ith input of g with all other coordinates of x falling into place as in the picture below. There are no “plugs” in this instance since all coordinates of x play a role.* 
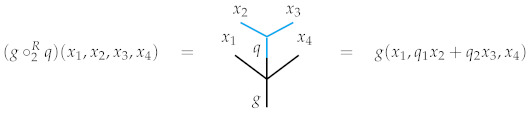

*These examples suggest the inner product notation is a convenient choice. Given N≥1 and k≤N and a point $x\in R^{N}$, let $x_{i,k}\in R^{k}$ denote the k-subtuple of x beginning at the ith coordinate:*

$$x_{i,k}:=\left(x_{i},\ldots ,x_{i+k-1}\right).$$

*Then given any point $x\in R^{n+m-1}$, the left and right composition maps may be written more succinctly as*

$$\begin{array}{cc} \left(p\circ_{i}^{L}f\right)\left(x\right) & =p_{i}f\left(x_{i,m}\right)\\ \left(g\circ_{i}^{R}q\right)\left(x\right) & =g(x_{1},\ldots ,x_{i-1},\langle q,x_{i,m}\rangle ,x_{i+m},\ldots ,x_{n+m-1}). \end{array}$$

*We will use this notation below and will always write $x_{i}$ in lieu of $x_{i,m}$ since the context will make it clear that $x_{i}$ must be an m-tuple. The boldface font is used to distinguish a tuple $x_{i}$ from a real number $x_{i}$. Finally, note that the maps $\circ_{i}^{L}$ and $\circ_{i}^{R}$ are continuous since f and g are continuous, and moreover that the associativity requirements in Equation (4) are analogous to those illustrated in Figure 2, so it is straightforward to verify they are satisfied. In particular, the zero functions appearing in the definition of $\circ_{i}^{L}$ simplify the situation greatly. For instance, several of associativity requirements follow from the simple fact that multiplying an input $x_{i}$ by a probability and then mapping the result to zero is the same as first mapping the input to zero and then multiplying that zero by a probability. So ${\mathrm{End}}_{R}$ is indeed a Δ-bimodule.*

Next, recall that the desired Leibniz rule in Equation (3) suggests the bimodule should be equipped with a notion of addition. This motivates the following definition.

**Definition** **4.** *Let O be an operad in a symmetric monoidal category C. An O-bimodule M is an*abelian O-bimodule*if each M(n) is an abelian monoid in C; that is, if for each n=1,2,… the following hold:*

*(i)*  *[associativity, commutativity] there is a morphism $\mu_{n}:M\left(n\right)\times M\left(n\right)\to M\left(n\right)$ in C such that $\mu_{n}\left(\mu_{n}\left(a,b\right),c\right)=\mu_{n}\left(a,\mu_{n}\left(b,c\right)\right)$ and $\mu_{n}\left(a,b\right)=\mu_{n}\left(b,a\right)$ for all a,b,c∈M(n),*
*(ii)*  *[identity] there is an element 1∈M(n) such that $\mu_{n}\left(1,a\right)=a=\mu_{n}\left(a,1\right)$ for all a∈M(n).*

As the primary example, consider ${\mathrm{End}}_{R}$ viewed as a Δ-bimodule as described in Example 3. For each n, define $\mu_{n}:{\mathrm{End}}_{R}\left(n\right)\times {\mathrm{End}}_{R}\left(n\right)\to {\mathrm{End}}_{R}\left(n\right)$ by pointwise addition, meaning that for each $f,g\in {\mathrm{End}}_{R}\left(n\right)$ we have $\mu_{n}\left(f,g\right)=f+g$ where (f+g)(x):=f(x)+g(x) for all $x\in R^{n}$. The identity element in ${\mathrm{End}}_{R}\left(n\right)$ is the constant map at zero. Moreover each $\mu_{n}$ is continuous and inherits associativity and commutativity from R. In this way, ${\mathrm{End}}_{R}$ is an abelian Δ-bimodule.

**Remark** **1.** *Notice that the *Δ*-bimodule composition maps $\circ_{i}^{L}$ and $\circ_{i}^{R}$ distribute over sums in the abelian *Δ*-bimodule ${\mathrm{End}}_{R}$. In other words, for all continuous functions $f,g\in {\mathrm{End}}_{R}\left(n\right)$ and for all probability distributions $q\in \Delta_{m}$,*

$$\begin{array}{c} \left(f+g\right)\circ_{i}^{R}q=f\circ_{i}^{R}q+g\circ_{i}^{R}q,\;1\leq i\leq n \end{array}$$

*and similarly for left composition $\circ_{i}^{L}$. This follows directly from pointwise addition.*

With this setup in mind, our desideratum in Equation (3) is now realized in the following definition.

**Definition** **5.** *Let O be an operad in a category C and let M be an abelian O-bimodule. A*derivation of O valued in *M is sequence of morphisms $\left\{d_{n}:O\left(n\right)\to M\left(n\right)\right\}$ in C satisfying*

(5) $$\begin{array}{c} d_{n+m-1}\left(p\circ_{i}q\right)=d_{n}p\circ_{i}^{R}q+p\circ_{i}^{L}d_{m}q \end{array}$$

*for all p∈O(n),q∈O(m) and for all 1≤i≤n.*

In the special case when O is a linear operad, this definition coincides with that given by Markl in [15]. In what follows, we omit the subscripts and simply write *d* instead of $d_{n}$. Now, suppose O=Δ is the operad of topological simplices and ${\mathrm{End}}_{R}$ is equipped with the structure of an abelian Δ-bimodule given above. Here is the picture to have in mind for Equation (5): 
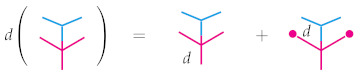


On the right-hand side we have used the “plug” notation introduced in Example 3, which can also be understood explicitly by evaluating *d* at a point $x\in R^{n+m-1}$,

$$\begin{array}{cc} d\left(p\circ_{i}q\right)\left(x\right) & =\left(dp\circ_{i}^{R}q\right)\left(x\right)+\left(p\circ_{i}^{L}dq\right)\left(x\right)\\ \; & =dp\left(x_{1},\ldots ,\langle q,x_{i}\rangle ,\ldots ,x_{n+m-1}\right)+p_{i}dq\left(x_{i}\right). \end{array}$$

Of particular interest is the behavior of a derivation $\left\{d:\Delta_{n}\to {\mathrm{End}}_{R}\left(n\right)\right\}$ when it is applied to a simultaneous composition of probability distributions. A derivation applied to the composite $\left(p\circ_{j}q\right)\circ_{i}r$ for probability distributions $p\in \Delta_{n},q\in \Delta_{m}$, and $r\in \Delta_{k}$ can be understood in a convenient picture when *q* and *r* are composed onto different leaves of *p*; that is, when 1≤i≤j−1 or i≥j+m. This follows straightforwardly from a repeated application of *d*. Indeed, by definition we have $d\left(\left(p\circ_{j}q\right)\circ_{i}r\right)=d\left(p\circ_{j}q\right)\circ_{i}^{R}r+\left(p\circ_{j}q\right)\circ_{i}^{L}dr$ and by applying the Leibniz rule again to the first summand, this is equal to $\left(dp\circ_{j}^{R}q+p\circ_{j}^{L}dq\right)\circ_{i}^{R}r+\left(p\circ_{j}q\right)\circ_{i}^{L}dr,$ which we can expand to obtain $\left(dp\circ_{j}^{R}q\right)\circ_{i}^{R}r+\left(p\circ_{j}^{L}dq\right)\circ_{i}^{R}r+\left(p\circ_{j}q\right)\circ_{i}^{L}dr$ since composition distributes over sums as noted in Remark 1. We will identify this function with the picture below in lieu of the cumbersome notation. 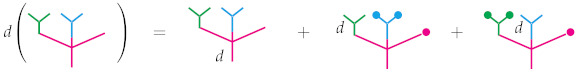


Importantly, the obvious generalization of the formula holds for any simultaneous composition $p\circ (q^{1},\ldots ,q^{n})$ for any $p\in \Delta_{n}$ and $q^{i}\in \Delta_{k_{i}}$ where i=1,…,n. This again follows directly from repeated applications of Equation (5), as illustrated below. 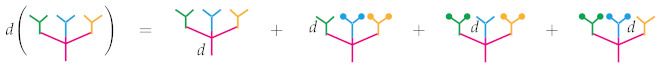


This is summarized in the following proposition.

**Proposition** **1.** *Let $p\in \Delta_{n}$ and $q^{i}\in \Delta_{k_{i}}$ for $n,k_{1},\ldots ,k_{n}\geq 1$ and let $\left\{d:\Delta_{n}\to {\mathrm{End}}_{R}\left(n\right)\right\}$ be a derivation of the operad of topological simplices. Then for any point $x\in R^{k_{1}+\cdots +k_{n}}$,*

$$d\left(p\circ \left(q^{1},\ldots ,q^{n}\right)\right)\left(x\right)=dp\left(\langle q^{1},x_{1}\rangle ,\cdots ,\langle q^{n},x_{n}\rangle \right)+\sum_{i=1}^{n}p_{i}dq^{i}\left(x_{i}\right).$$

Finally, the main result follows.

**Theorem** **3.** *Shannon entropy defines a derivation of the operad of topological simplices, and for every derivation of this operad there exists a point at which it is given by a constant multiple of Shannon entropy.*

**Proof.**  For each n≥1 define $d:\Delta_{n}\to {\mathrm{End}}_{R}\left(n\right)$ by p↦dp where dp(x)=H(p) is constant for all $x\in R^{n}$. Then, *d* is continuous since *H* is continuous. Moreover, if $p=\left(p_{1},\ldots ,p_{n}\right)\in \Delta_{n}$ and $q=\left(q_{1},\ldots ,q_{m}\right)\in \Delta_{m}$ are probability distributions, then for any $x\in R^{m+n-1}$ and 1≤i≤n, we have

$$\begin{array}{cc} d\left(p\circ_{i}q\right)\left(x\right)=H\left(p\circ_{i}q\right) & =-\left(\sum_{k=1}^{i-1}p_{k}\log p_{k}+p_{i}\sum_{k=1}^{m}q_{k}\log\left(p_{i}q_{k}\right)+\sum_{k=i+1}^{n}p_{k}\log p_{k}\right)\\ \; & =-\left(\sum_{k=1}^{i-1}p_{k}\log p_{k}+p_{i}\log p_{i}\sum_{k=1}^{m}q_{k}+p_{i}\sum_{k=1}^{m}q_{k}\log q_{k}+\sum_{k=i+1}^{n}p_{k}\log p_{k}\right)\\ \; & =-\left(\sum_{k=1}^{n}p_{k}\log p_{k}+p_{i}\sum_{k=1}^{m}q_{k}\log q_{k}\right)\\ \; & =H\left(p\right)+p_{i}H\left(q\right)\\ \; & =\left(dp\circ_{i}^{R}q+p\circ_{i}^{L}dq\right)\left(x\right), \end{array}$$

where the last line follows since $\left(dp\circ_{i}^{R}q\right)\left(x\right)$ is computed by evaluating the function dp at some point, and this function is assumed to be constant at H(p).

Conversely, suppose $\left\{d:\Delta_{n}\to {\mathrm{End}}_{R}\left(n\right)\right\}$ is a derivation. For each n≥1 define a function $F:\Delta_{n}\to R$ by F(p)=dp(0) where $0=\left(0,\ldots ,0\right)\in R^{n}$. Then *F* is continuous since *d* is continuous, and Proposition 1 further implies that

$$\begin{array}{cc} F(p\circ \left(q^{1},\ldots ,q^{n}\right)) & =d\left(p\circ \left(q^{1},\ldots ,q^{n}\right)\right)\left(0\right)\\ \; & =dp\left(\langle q^{1},0_{1}\rangle ,\ldots ,\langle q^{n},0_{n}\rangle \right)+\sum_{i=1}^{n}p_{i}dq^{i}\left(0_{i}\right)\\ \; & =dp\left(0\right)+\sum_{i=1}^{n}p_{i}dq^{i}\left(0\right)\\ \; & =F\left(p\right)+\sum_{i=1}^{n}p_{i}F\left(q^{i}\right). \end{array}$$

From the Faddeev–Leinster result in Theorem 1, it follows that dp(0)=F(p)=cH(p) for some c∈R. □

Notice that the important Equation (2) mentioned in the introduction is obtained as a corollary. Indeed, if for each n≥1 the map $d:\Delta_{n}\to {\mathrm{End}}_{R}\left(n\right)$ is defined to be constant at entropy p↦dp≡H(p), then *d* is a derivation by Theorem 3 and so Proposition 1 yields the following by evaluating $d(p\circ \left(q^{1},\ldots ,q^{n}\right))$ at any point.

**Corollary** **1.** *Let $p\in \Delta_{n}$ and $q^{i}\in \Delta_{k_{i}}$ with 1≤i≤n. Then*

$$H\left(p\circ \left(q^{1},\ldots ,q^{n}\right)\right)=H\left(p\right)+\sum_{i=1}^{n}p_{i}H\left(q^{i}\right).$$

As a closing remark, Faddeev’s characterization of entropy in Theorem 1 can be reexpressed using the language of category theory and operads as in [1], (Theorem 12.3.1). We have omitted this language here but invite the reader to explore the full category theoretical story in Chapter 12 of Leinster’s book.

## Data Availability

Not applicable.
